# Ubiquitome analysis reveals the involvement of lysine ubiquitination in the spermatogenesis process of adult buffalo (*Bubalus bubalis*) testis

**DOI:** 10.1042/BSR20193537

**Published:** 2020-06-16

**Authors:** Yu-lin Huang, Peng-fei Zhang, Zhen Hou, Qiang Fu, Ming-xing Li, De-lun Huang, Ting-xian Deng, Yang-qing Lu, Xian-wei Liang, Ming Zhang

**Affiliations:** 1 College of Life Science and Technology, Guangxi University, Nanning, Guangxi, China; 2 State Key Laboratory for Conservation and Utilization of Subtropical Agro-Bioresources, Animal Reproduction Institute, Guangxi University, Nanning, Guangxi, China; 3 Department of Cell and Genetics, College of Basic Medicine, Guangxi University of Chinese Medicine, Nanning 530004, Guangxi, China; 4 Key Laboratory of Buffalo Genetics, Breeding and Reproduction Technology, Ministry of Agriculture and Guangxi, Buffalo Research Institute, Chinese Academy of Agricultural Sciences, Nanning, Guangxi, China

**Keywords:** buffalo testis, LC-MS/MS, lysine ubiquitination, post translational modification, proteomics, spermatogenesis

## Abstract

Protein ubiquitination, a major and conserved post-translational modification, is known to play a critical regulatory role in many biological processes in eukaryotes. Although several ubiquitinated proteins have been found in buffalo (*Bubalus bubalis*) testis in our previous studies, large-scale profiling of buffalo testis ubiquitome has not been reported to date. In the present study, we first identified a global profiling of lysine ubiquitination of adult buffalo testis using a highly sensitive LC-MS/MS coupled with immune-affinity enrichment of ubiquitinated peptides. In total, 422 lysine ubiquitination sites were identified in 262 proteins in adult buffalo testis tissue. Bioinformatics analysis showed that the ubiquitinated proteins are involved in a variety of biological processes and diverse subcellular localizations. Kyoto Encyclopedia of Genes and Genomes (KEGG) pathway and protein interaction network analysis indicated that proteasome, glycolysis/gluconeogenesis and gap junction pathways are modulated by protein ubiquitination in testis. Besides, 44 ubiquitinated proteins may involve in spermatogenesis according to the SpermatogenesisOnline database, of which, the ubiquitination of HSPA2 and UCHL1 were confirmed by Immunoprecipitation (IP)/Western blot analysis. Taken together, these data provide a global view of ubiquitome in buffalo testis for the first time, and serve as an important resource for exploring the physiological role especially spermatogenesis of lysine ubiquitination in testis in mammals.

## Introduction

Ubiquitin (Ub) is a small 76-amino-acid protein that is highly conserved in eukaryotic organisms. It is conjugated to the ε-amino group of Lysines (Lys) and always found in both cytosol and nucleus of eukaryotic cells [1]. The ubiquitin–proteasome system (UPS) is facilitated to protein degradation and consisted of three major enzymes: ubiquitin-activating enzymes (E1), ubiquitin-conjugating enzymes (E2), and ubiquitin ligase enzymes (E3) [2]. Ubiquitylation is involved in the regulation of many other cellular processes such as DNA replication [3], DNA damage repair [4,5], innate immune signaling, and receptor endocytosis [6,7]. Besides, over the years, a growing number of studies have indicated that ubiquitin system plays a vital role in spermatogenesis and fertilization [8,9].

The testis is an important sexual organ in male mammals. Specially, testis is the site of spermatogenesis, a complicated and dynamic process in which the male germ cell metamorphoses into mature spermatozoa. During mammalian spermatogenesis, three major events including spermatocytogenesis, meiosis, as well as spermiogenesis occurred in the seminiferous tubules. The spermatogonia must undergo meiosis and the round spermatids have to abandon redundant cytoplasm materials, in other words, many proteins and organelles are degraded, and the ubiquitin-proteasome pathway facilitates to form the highly condensed sperm in this process [10]. Kopanja et al. demonstrated that E3 ubiquitin ligase CUL4A is essential for spermatogenesis and the Cul4A knockout male mice are infertile [11]. The E2 ubiquitin-conjugating enzyme UBE2J1 is also required for spermiogenesis and the male UBE2J1 knockout mice are sterile with defects in flagella and cytoplasm removal in sperm cells [12]. In addition, deletion of deubiquitinating enzyme CYLD reduces the early wave of germ cell apoptosis and leads to impaired spermatogenesis [13]. In our previous study, we found E3 ubiquitin-protein ligase TRIM36 was expressed highly in puberty of buffalo testicular seminiferous tubules, and bioinformatics analysis indicated that TRIM36 may involve in acrosome reaction and regulation of cell cycle [14]. Hence, the identification of related ubiquitinated proteins is of great significance for the study of the function of ubiquitination in spermatogenesis.

Mass spectrometry (MS)-based proteomics approaches are appropriate to acquire large-scale ubiquitinated protein profile. For example, 110 precise ubiquitination sites were found in yeast [15], and thousands of targets were mapped in mammalian cells [16–18]. Nevertheless, due to the need for enrichment of ubiquitinated proteins in the methods, only a relatively small number of ubiquitinated sites were identified in these studies. Recently, a preferable method based affinity purification of ubiquitinated peptides combined with highly sensitive LC-MS/MS was used for comprehensively ubiquitylation site identification. As is known to all, after the ubiquitinated proteins were digested by trypsin, the diglycine (di-Gly) remnant derived from the two C-terminal glycine residues of ubiquitin maintained covalently linked to the modified lysines. Accordingly, a di-Gly-lysine specific antibody [19] enables the affinity capture of ubiquitinated peptides. The distinct mass shift (114.0429 Da) caused by the di-Gly remnant should be able to identify and precisely locate the ubiquitination sites based on peptide fragment masses [20]. Several researchers have employed the newly technique to process in-depth ubiquitin-modified proteome analyses and discover abundant ubiquitination sites in mammalian cells and tissues [19–22]. However, the adaptability of this method to mammalian testis has not been tested.

Buffalo (*Bubalus bubalis*) is of considerable economic and biological interest especially in the southern region in China, but its reproductive efficiency is low due to the delay onset of puberty. In buffalo, the approximate duration of spermatogenesis is 38 days, which represents the shortest period reported in the large domestic animals up to now [23]. Approximately 63% of all possible germ cells disappear from the seminiferous epithelium theoretically during spermatogenesis in buffalo [24]. Thus, elucidation of the ubiquitome in buffalo testis is important for understanding the role of the UPS in regulating spermatogenesis and fertilization in this ruminant species. In the present study, we performed a global profiling of the ubiquitome of adult buffalo testis using integrated proteomic techniques in which all ubiquitylated peptides are directly enriched from peptide mixtures with a high-affinity anti-di-Gly-lys-specific antibody. Overall, a total of 422 lysine ubiquitination sites were identified in 262 proteins in adult buffalo testis, regulating a wide range of biological processes especially metabolic and reproductive processes. The results provide an overall view of ubiquitome of buffalo testis and an abundant dataset for exploring the physiological role of lysine ubiquitination in testis in mammals.

## Materials and methods

### Sample preparation and protein extraction

All swamp buffalo (*Bubalus bubalis*) testes (age 3–5 years) were collected from a local commercial slaughterhouse and transported to the lab in sterile isotonic saline within 4 h and the tunica vaginalis and epididymis were removed. The three collected testes were divided into three separate samples to obtain three biological replicates. The weight and length of each testis were recorded and the detailed data listed in Supplementary Table S1. All procedures involving animal treatment and collection of testes to be used in the study were based on the Guiding Principles for animal use as described by the Council for International Organizations of Medical Sciences (CIOMS) and approved by the Animal Experimentation Ethics Committee of Guangxi University, Nanning, China. The adult buffalo testicular tissue was dissected into small pieces and ground to powder in liquid nitrogen and then transferred to a 5-ml centrifuge tube. After that, four volumes of lysis buffer (8 M urea, 2 mM EDTA, 1% Protease Inhibitor Cocktail and 50 μM PR-619) were added to the cell powder, followed by sonication three times on ice using a high-intensity ultrasonic processor (Scientz, Ningbo, Zhejiang, China). The remaining debris was removed by centrifugation at 12000 ***g*** at 4°C for 10 min. Finally, the supernatant was collected and the protein concentration was determined with BCA kit (Solarbio, Beijing, China) according to the manufacturer’s instructions.

### Trypsin digestion

For digestion, the protein solution was reduced with 5 mM dithiothreitol (DTT) for 30 min at 56°C and alkylated with 11 mM iodoacetamide for 15 min at room temperature in the dark. The protein sample was then diluted by adding 100 mM NH_4_HCO_3_ to urea concentration less than 2 M. Finally, trypsin was added to the diluted protein sample at 1:50 trypsin-to-protein mass ratio for the first digestion overnight and 1:100 trypsin-to-protein mass ratio for a second digestion for 4 h.

### Affinity enrichment of the ubiquitinated peptides

To enrich ubiquitinated peptides, tryptic peptides dissolved in NETN buffer (100 mM NaCl, 1 mM EDTA, 50 mM Tris/HCl, 0.5% NP-40, pH 8.0) were incubated with pre-washed anti-K-ε-GG beads (Lot number PTM-1104, PTM Biolabs, Hangzhou, China) at 4°C overnight with gentle shaking. Then the beads were washed four times with NETN buffer and twice with ddH_2_O. The bound peptides were eluted from the beads with 0.1% trifluoroacetic acid (TFA) thrice. Finally, the eluted fractions were combined and vacuum-dried. For LC-MS/MS analysis, the resulting peptides were desalted with C18 ZipTips (Millipore) according to the manufacturer’s instructions.

### LC-MS/MS analysis

The enriched peptides were dissolved in solvent A (0.1% formic acid), directly loaded on to a reversed-phase analytical column (15-cm length, 75 μm i.d.). The gradient comprised an increase from 10 to 22% solvent B (0.1% formic acid in 90% acetonitrile) over 40 min, 22 to 35% in 12 min and climbing to 80% in 4 min, then holding at 80% for the last 4 min, all at a constant flow rate of 700 nl/min on an EASY-nLC 1000 UPLC system. The peptides were subjected to NSI source followed by tandem mass spectrometry (MS/MS) in Orbitrap Fusion™ (Thermo) coupled online to the UPLC. The electrospray voltage applied was 2.0 kV. The m/z scan range was 350–1550 for full scan, and intact peptides were detected in the Orbitrap at a resolution of 60000. A data-dependent procedure that alternated between one MS scan followed by 20 MS/MS scans with 15.0 s dynamic exclusion. Automatic gain control (AGC) was set at 5E4.

The MS proteomics data have been deposited in the ProteomeXchange Consortium via the PRIDE [25] partner repository with the dataset identifier PXD012231.

Username: reviewer50874@ebi.ac.uk
Password: mtGAMTcX

### Database search

The resulting MS/MS data were processed using Maxquant search engine (v.1.5.2.8). Tandem mass spectra were searched against Proteomes *Bos taurus (*24215 sequences) database concatenated with reverse decoy database. Trypsin/P was specified as cleavage enzyme allowing up to four missing cleavages. The mass tolerance for precursor ions was set as 20 ppm in first search and 5 ppm in main search, and the mass tolerance for fragment ions was set as 0.02 Da. Carbamidomethyl on Cys was specified as fixed modification and Gly–Gly modification for lysines and oxidation on Met were specified as variable modifications. FDR was adjusted to <1% and minimum score for modified peptides was set as >40. The site localization probability was set as >0.75.

### Bioinformatics analysis

The Motif-X software [26] was used to analyze the model of sequences with amino acids in specific positions of ubiquityl-21-mers (10 amino acids upstream and downstream of the site) in all protein sequences. The *B. taurus* proteome was used as the background database, the setting parameters were: occurrences = 20, Bonferroni corrected *P*-value =0.005, and the other parameters were set with default. Secondary structures of all proteins were predicted by NetSurfP software [27]. The mean secondary structure probabilities of the modified lysine residues were compared with those of the control residues for all ubiquitinated proteins identified in the present study. The domain annotation was performed with InterProScan on the InterPro domain database (http://www.ebi.ac.uk/interpro/) [28] and the protein domains with a corrected *P*-value <0.05 were considered significant. WoLF PSORT [29] was used to predict the subcellular localization. Gene Ontology (GO) annotation proteome was derived from the UniProt-GOA database (http://www.ebi.ac.uk/GOA/) [30] and the lysine ubiquitination proteins were classified by GO annotation involving three categories: biological processes, molecular functions, and cellular components. The SpermatogenesisOnline database (https://mcg.ustc.edu.cn/bsc/spermgenes/documentation.php) [31] was used to analyze and verify spermatogenesis-related process terms. The Kyoto Encyclopedia of Genes and Genomes (KEGG) database [32] was used to perform pathway analysis. Functional annotation tool of DAVID bioinformatics resources 6.8 (https://david.ncifcrf.gov) [33] was used to identify GO term and KEGG pathway. Protein–protein interaction network among the surveyed proteins was retrieved from STRING database (version 10.5) with a confidence score of at least 0.7, and the interaction network was visualized in Cytoscape software [34].

### Immunohistochemistry analysis

Immunohistochemistry was performed on 4% paraformaldehyde-fixed buffalo testes. The paraffin-embedded testicular sections were dewaxed and dehydrated, and endogenous peroxidase activity was quenched by methanol containing 3% H_2_O_2_ for 30 min. Sections were subjected to antigen retrieval in 0.01 M sodium citrate buffer (pH 6.0) using microwave heating. The sections were then incubated in blocking solution (5% BSA) for 2 h at room temperature and incubated overnight at 4°C with primary antibody against UCHL1 (7863-1004, AbD, Oxford, U.K.) at a dilution of 1:200. After washing twice in PBS-Tween-20, the sections were incubated with secondary antibody labeled with FITC (ab6717, abcam, 1:200) for 1 h at room temperature, and then cover-slipped in Prolong Gold antifade reagent with DAPI (Life Technologies) and kept in the dark until photographed using an Olympus IX73 inverted fluorescence microscope (Olympus, Tokyo, Japan). Negative control was incubated in blocking solution without the primary antibody.

### Immunoprecipitation and Western blot analysis

To confirm the ubiquitination of proteins utilizing the K-ε-GG antibody, we performed immunoprecipitation (IP) and Western blot analysis. The lysed cell extracts were immunoprecipitated with anti-ubiquitin antibody (ab105015; Abcam, Cambridge, MA, U.S.A.) or rabbit IgG (ab205718, Abcam) using protein A+G agarose (Beyotime, Shanghai, China), after shaking at 4°C for 3 h, the supernatant was removed carefully by centrifugation at 1000 ***g*** for 5 min and the precipitate was washed for five times by PBS buffer (8 mM Na_2_HPO_4_, 1.5 mM KH_2_PO_4_, 135 mM NaCl, and 2.7 mM KCl), then 20 μl of 1× SDS/PAGE loading buffer (CWBIO, Beijing, China) was added into the precipitate and used for electrophoresis after boiling at 100°C for 5 min. After electrophoresis, the SDS/PAGE gel was transferred on to a polyvinylidene difluoride (PVDF) membrane by a semidry Western blotting system (Bio-Rad). The membrane was blocked with 5% nonfat milk in TBST solution for 2 h at room temperature and then incubated overnight at 4°C with primary antibody against UCHL1 (7863-1004, AbD, Oxford, U.K.) and HSPA2 (bs-18080R, Bioss Biotechnology Inc., China) at a dilution of 1:1000. Membranes washed with TBST buffer three times were incubated with horseradish peroxidase-conjugated secondary antibodies (CWBIO, Beijing, China) in TBST buffer for 1.5 h at room temperature. Bands were visualized with an ECL detection kit.

## Results

### Proteome-wide analysis of lysine-ubiquitinated sites and proteins in buffalo testis

**Figure 1:**
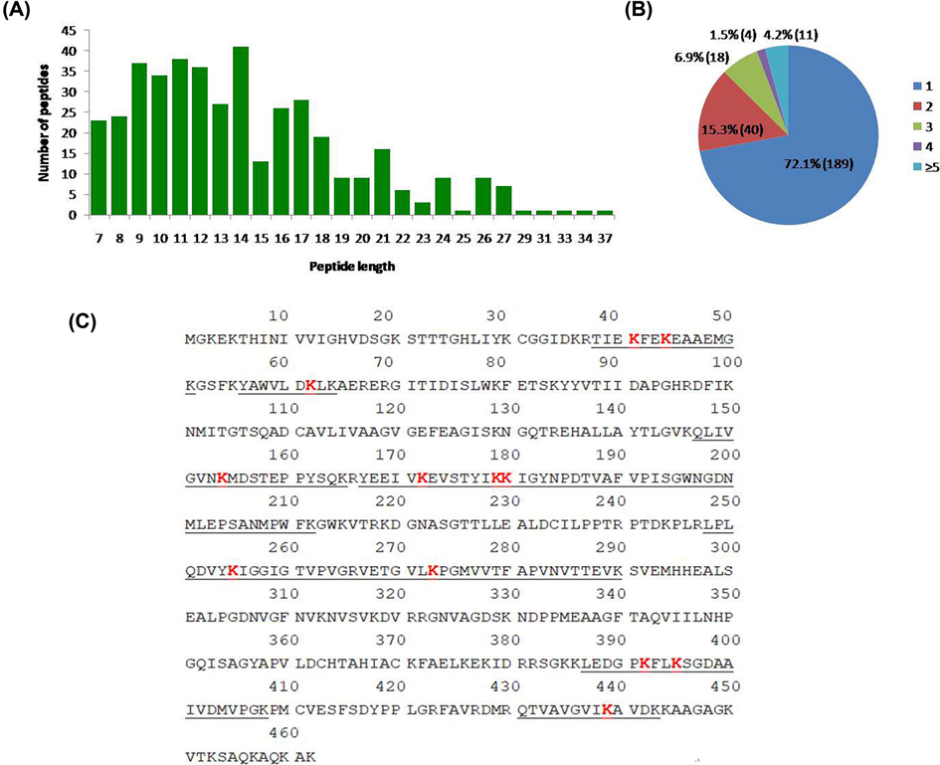
Identification of lysine ubiquitinated sites in buffalo testis (**A**) Peptide length distribution of the ubiquitinated peptides. (**B**) Distribution of the number of lysine ubiquitination sites in the identified ubiquitinated peptides. (**C**) Twelve lysine ubiquitination sites present in EEF1A1. Red sequences indicate the lysine ubiquitinated sites. Underlined sequences indicate the identified ubiquitinated peptides. Abbreviation: EEF1A1, elongation factor 1-α 1.

In our previous proteomic study of buffalo testicular seminiferous tubules, we identified many ubiquitin enzymes including USP47, USP14, UCHL1, UCHL5, UBE2K, UBE2V2, UFC1, and UBA1 (Supplementary Table S2) [14]. In order to study the roles of ubiquitinated proteins in buffalo testis, we performed proteome-wide analysis of lysine ubiquitination sites and proteins in normal adult buffalo testis. A total of 422 lysine ubiquitination sites were identified unambiguously, corresponding to 262 lysine ubiquitinated proteins (Supplementary Table S3). The length of most ubiquitinated peptides ranged from 7 to 34 amino acids, which is consistent with the property of tryptic peptides (Figure 1A).

We sorted the ubiquitinated proteins according to the number of ubiquitination sites, as shown in Figure 1B. Among these ubiquitinated proteins, 72.1% (189) contained a single ubiquitination site, and 15.3% (40) contained two ubiquitination sites. It is noteworthy that 4.2% (11) of proteins contained five or more lysine ubiquitination sites and f had at least nine sites (Supplementary Table S4). Elongation factor 1-α 1 (EEF1A1) protein was the most intensely ubiquitinated protein, bearing 12 different lysine ubiquitination sites (Figure 1C). Some ubiquitinated proteins were determined in buffalo testis in our previous studies, but the detailed ubiquitination sites remain vague. In the present study, USP4 protein was found to be ubiquitinated at K837, UBE2N protein was found to be ubiquitinated at K92, and the protein USP5 was found to be ubiquitinated at K163, K357, K360, K406, K423, K468, K574, K575, K586, K743, and K793. The representative MS/MS spectra of ubiquitinated peptides from USP5 protein were shown in Supplementary Figure S1.

### Motif analysis around identified ubiquitinated sites

**Figure 2:**
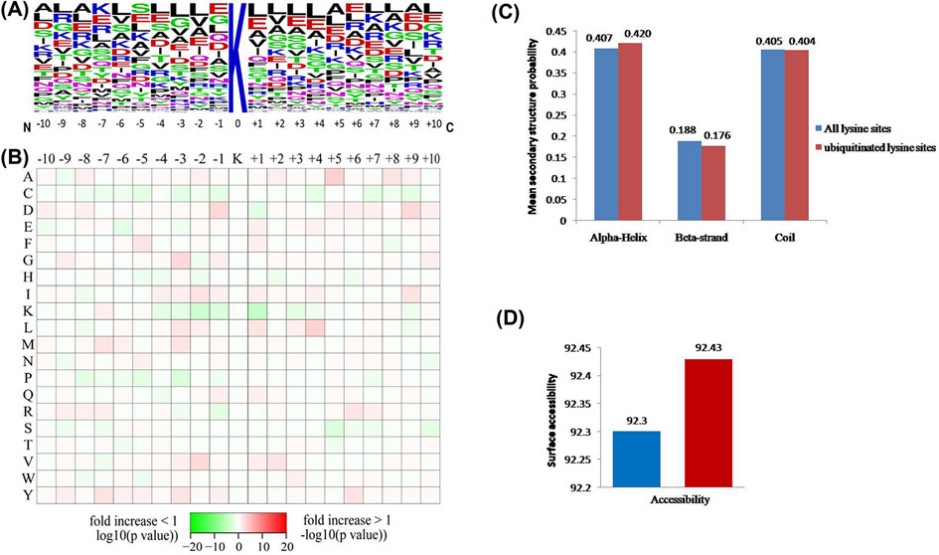
The properties of lysine ubiquitination sites (**A**) Probable sequence motifs for ±10 amino acids around the lysine ubiquitination sites. The central K stands for the ubiquitinated lysine and the height of each letter corresponding to the frequency of the amino acid residue in that position. (**B**) Heat map showing the relative frequencies of amino acids in specific positions. Red and green respectively indicate the amino acid is significantly enriched or reduced near the ubiquitinated site. (**C**) Probable distribution of secondary structures containing lysine ubiquitination sites. (**D**) Predicted surface accessibility of the ubiquitinated sites. Ubiquitinated lysine sites were in red and all lysine sites were in green.

We further performed motif analysis of the sequences around the identified ubiquitinated sites using motif-x program to reveal the distribution of the surrounding amino acids. No significant sequence recognition motif was identified among the ubiquitination sites (Figure 2A,B), which was consistent with previous observations in *Arabidopsis* and human cells [17,19,21,35]. Our results showed that there was a subtle enrichment for specific residues at some positions, such as leucine (L) at +1, +3, and +4 positions, valine (V) at −2 and +2 positions, alanine (A) at +5 position, glycine (G) at −3 position, and aspartate (D) at −1 position. It is probably that the motifs were affected by the sequence bias of the antibody enrichment technique employed here, and a similar discovery has been noted by employing two antibodies for enrichment of ubiquitinated peptides [22].

In order to investigate the properties of ubiquitylation sites in more detail, the local secondary structures of ubiquitylated proteins were investigated (Figure 2C). The results showed that 59.6% of the ubiquitylated sites were located in regions of ordered secondary structure. Among them, 42% sites were located in α-helix and 17.6% sites in β-strand. The rest of 40.4% were located in disordered regions of the proteins (coil). Nevertheless, because of the similarity of distribution pattern of secondary structures between ubiquitylated sites and all lysine sites (*P*>0.05), it seems that there is no tendency of ubiquitination in proteins of buffalo testis. In addition to ordered regions, we further evaluated the surface accessibility of the ubiquitylated lysine sites. The results indicated that 92.43% of the ubiquitylated lysine sites were exposed to protein surface, compared with 92.3% of all lysine sites (*P*=0.036) (Figure 2D). Although the difference is subtle, the surface property of proteins is likely to be changed by lysine ubiquitination.

### Functional classification and cellular localization of ubiquitinated proteins

**Figure 3:**
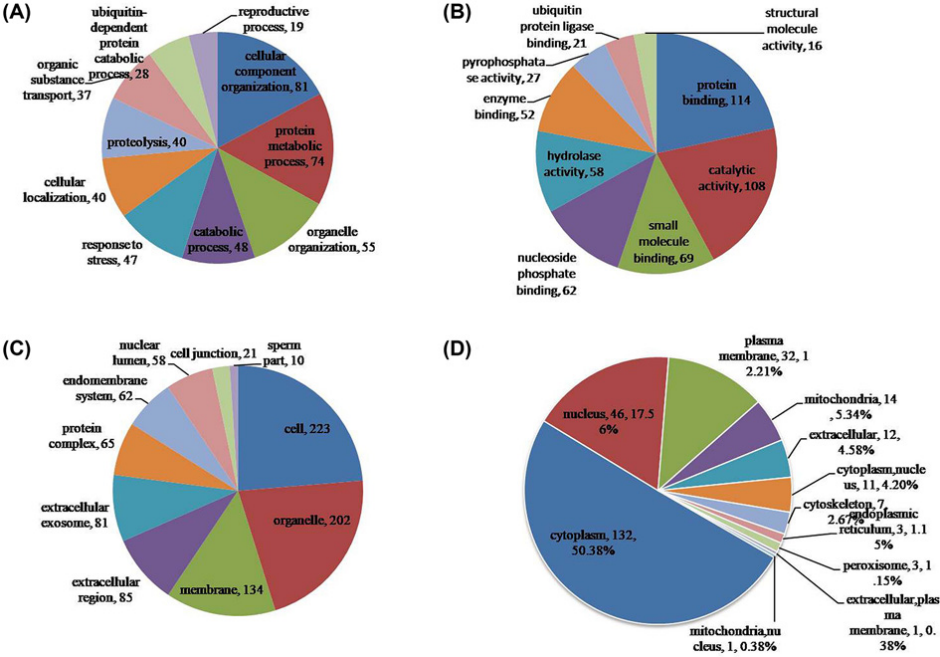
Functional classification of ubiquitinated proteins in buffalo testis (**A**) Classification of the ubiquitinated proteins based on biological process. The ubiquitinated proteins were involved in many biological processes, large group of them were related to cellular component organization (81), followed by protein metabolic process (74), organelle organization (55), catabolic process (48) and response to stress (47), others were assigned to ubiquitin-dependent protein catabolic process (28) and reproductive process (19). (**B**) Classification of the ubiquitinated proteins based on molecular function. Most of ubiquitinated proteins were related to protein binding (114) and catalytic activity (108), others were assigned to small molecule binding (69), nucleoside phosphate binding (62), hydrolase activity (58), and ubiquitin protein ligase binding (21). (**C**) Classification of the ubiquitinated proteins based on cellular component. Most of ubiquitinated proteins were located in cell (223), organelle (202) and membrane (134), others were distributed in extracellular region (85), extracellular exosome (81), protein complex (65), endomembrane system (62), nuclear lumen (58), cell junction (21) and sperm part (10). (**D**) Subcellular localization of the ubiquitinated proteins. The largest group of ubiquitinated proteins is associated with cytoplasm (132, 50.38%), the other four subcellular components with active ubiquitination were nucleus (46, 17.56%), plasma membrane (32, 12.21%), mitochondria (14, 5.34%), and extracellular (12, 4.58%).

To better understand the ubiquitome in buffalo testis, the identified ubiquitinated proteins were annotated with GO annotation. All level GO terms were used to classify the proteins into three categories: biological process, cellular component, and molecular function (Figure 3A–C, Supplementary Table S5). The results showed that large group of ubiquitinated proteins were related to cellular component organization (81), followed by protein metabolic process (74), organelle organization (55), catabolic process (48) and response to stress (47), moreover, others were assigned to ubiquitin-dependent protein catabolic process (28) and reproductive process (19) (Figure 3A). Most of ubiquitinated proteins for molecular function classification were related to protein binding (114) and catalytic activity (108) (Figure 3B), suggesting that massive ubiquitinated proteins may be enzymatic proteins in the present study. Besides, others were assigned to small molecule binding (69), nucleoside phosphate binding (62), hydrolase activity (58), and ubiquitin protein ligase binding (21).

In the cellular component category, most of ubiquitinated proteins were located in cell (223), organelle (202) and membrane (134), others were distributed in extracellular region (85), extracellular exosome (81), protein complex (65), endomembrane system (62), nuclear lumen (58), cell junction (21), and sperm part (10) (Figure 3C). In order to elaborately subdivide the cells into functionally distinct membrane bound compartments, we further carried out subcellular localization analysis using WoLF PSORT software. The results revealed that the largest group of ubiquitinated proteins in buffalo testis is associated with cytoplasm (132, 50.38%), the other four subcellular components with active ubiquitination were nucleus (46, 17.56%), plasma membrane (32, 12.21%), mitochondria (14, 5.34%), and extracellular (12, 4.58%) (Figure 3D, Supplementary Table S6).

### Functional enrichment analysis of ubiquitinated proteins

**Figure 4:**
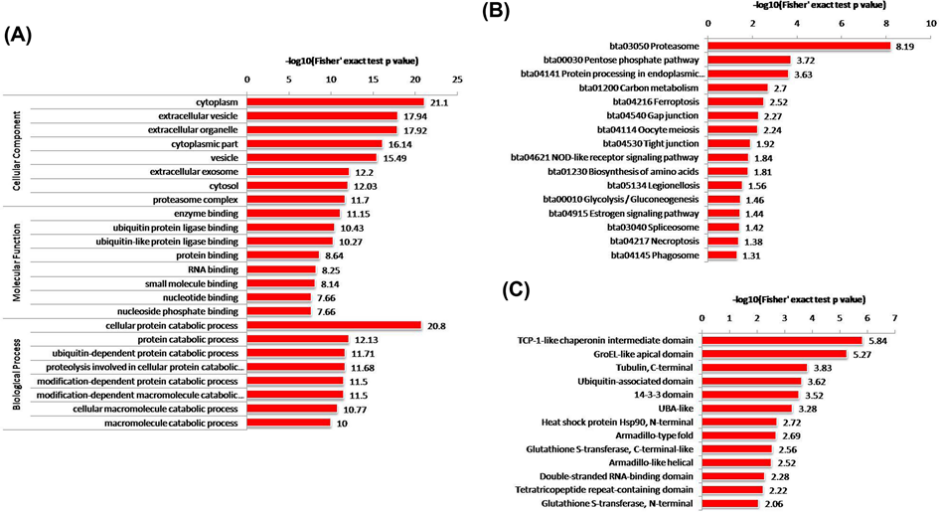
Enrichment analysis of the ubiquitinated proteins in buffalo testis (**A**) GO enrichment analysis of the identified ubiquitinated proteins in terms of cell component, molecular function, and biological process. (**B**) KEGG pathway enrichment analysis of the identified ubiquitinated proteins. (**C**) Protein domain enrichment analysis of the identified ubiquitinated proteins.

All ubiquitinated proteins were subjected to enrichment analysis to further elucidate the characterization in buffalo testis. GO enrichment analysis indicated that the ubiquitinated proteins were significantly enriched in cellular protein catabolic process, which is consistent with the observation that proteins with enzyme binding and ubiquitin protein ligase binding have a higher tendency to be ubiquitinated. In agreement with the subcellular localization analysis, the ubiquitinated proteins were significantly enriched in cytoplasm and extracellular vesicle based on the cellular component enrichment (Figure 4A, Supplementary Table S7).

To better understand the functions of these ubiquitinated proteins and how they affect buffalo testis, KEGG pathway and enrichment analysis were performed. The results showed that many ubiquitinated proteins were highly enriched in proteasome, pentose phosphate pathway, gap junction, tight junction, glycolysis/gluconeogenesis, and spliceosome, etc (Figure 4B, Supplementary Table S8). Of which, most enrichment pathways were related to metabolism, genetic information processing, and cellular processes. Additionally, we analyzed the distribution of ubiquitination sites on proteins and found correlation of ubiquitination sites in domains by protein domain enrichment. A total of 145 proteins have lysine-ubiquitinated sites within functional domains, and in total of 87 domains have at least one ubiquitinated site identified (Figure 4C, Supplementary Table S9). Several domains in proteins such as TCP-1-like chaperonin intermediate domain, GroEL-like apical domain, tubulin (C-terminal), and ubiquitin-associated domain were highly enriched. It is possible that the structure of particular domains may be related to the function of ubiquitination.

### Protein interaction networks of ubiquitinated proteins in buffalo testis

**Figure 5:**
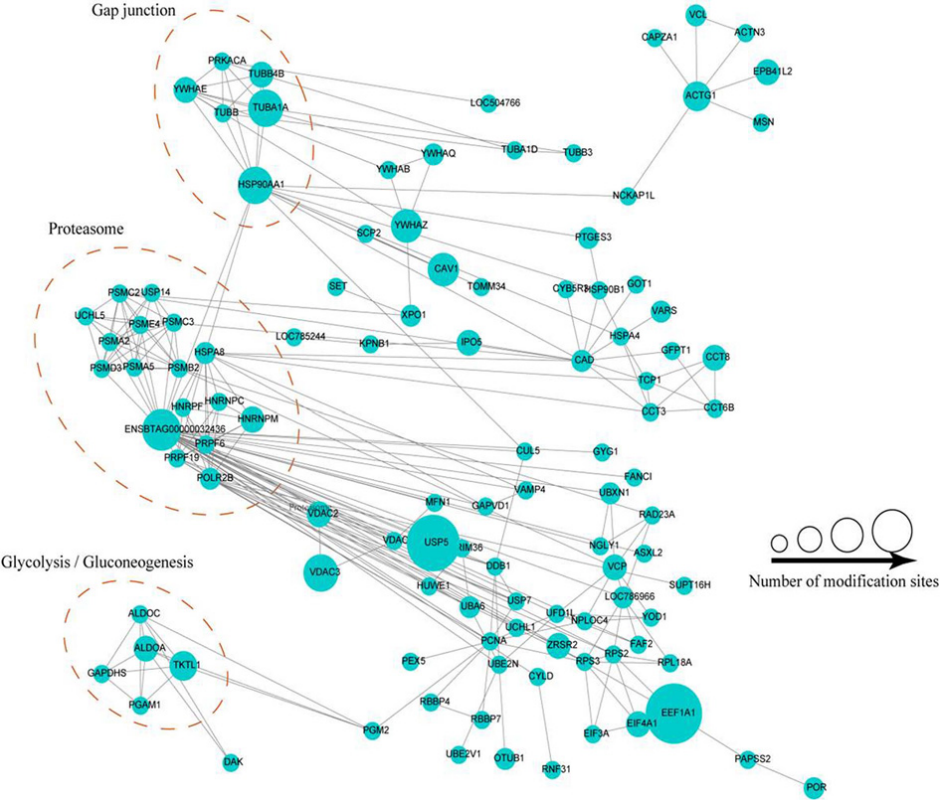
The whole protein–protein interaction network of the identified ubiquitinated proteins in buffalo testis Interaction network of ubiquitinated proteins involved in proteasome, gap junction, and glycolysis/gluconeogenesis pathway. The bubble size represents the number of ubiquitination sites.

**Figure 6:**
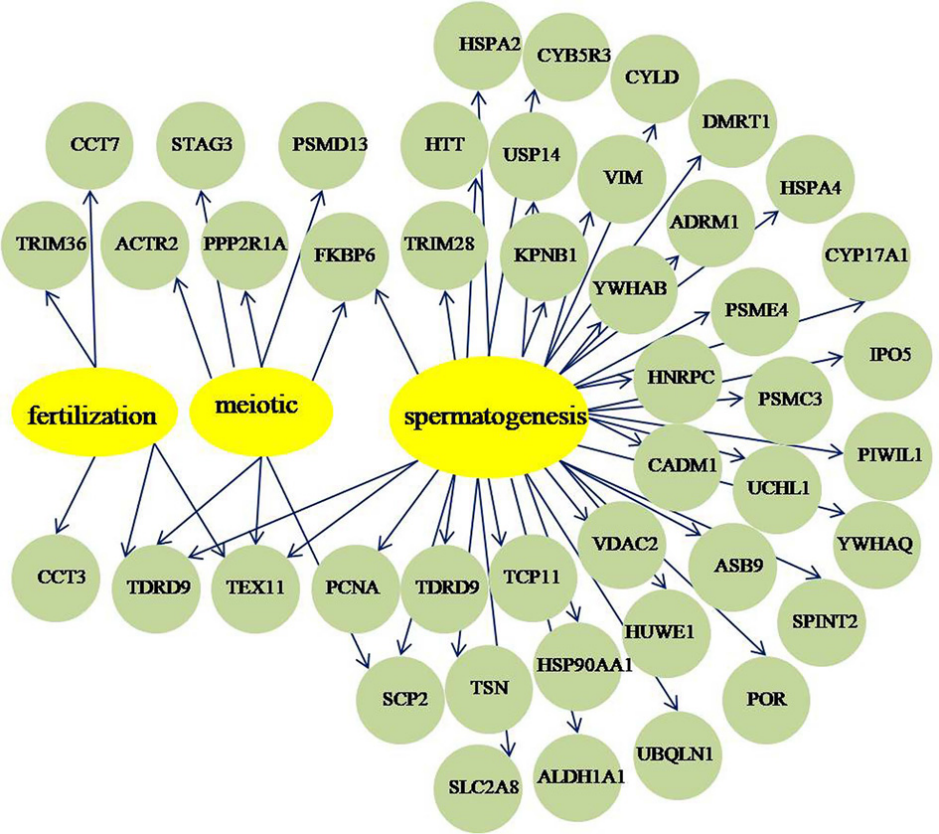
Ubiquitinated proteins involved in spermatogenesis, meiosis, and fertilization processes The yellow circles represent biological processes, and the green circles represent the involved ubiquitinated proteins.

To understand how the ubiquitinated proteins might interact with diverse pathways, we constructed the protein–protein interaction networks for all ubiquitinated proteins using STRING database and Cytoscape software. A large network containing 117 ubiquitinated proteins, and three highly interconnected clusters of ubiquitinated proteins were retrieved according to the algorithm of Cytoscape software (Figure 5, Supplementary Table S10). The first cluster consists of six ubiquitinated proteins involved in gap junction, the second cluster consists of seventeen ubiquitinated proteins involved in proteasome, and the third cluster consists of five ubiquitinated proteins associated with glycolysis/gluconeogenesis. The results suggested that these three pathways all displayed dense protein interaction networks and the physiological interactions may lead to their coordination and cooperation in buffalo testis. Interestingly, we found 44 lysine ubiquitinated proteins with known functions may important for meiotic, fertilization, and spermatogenesis through the network of testis-associated biological processes and the SpermatogenesisOnline database (Figure 6, Supplementary Table S11).

### Confirmation of the ubiquitination of certain proteins

**Figure 7:**
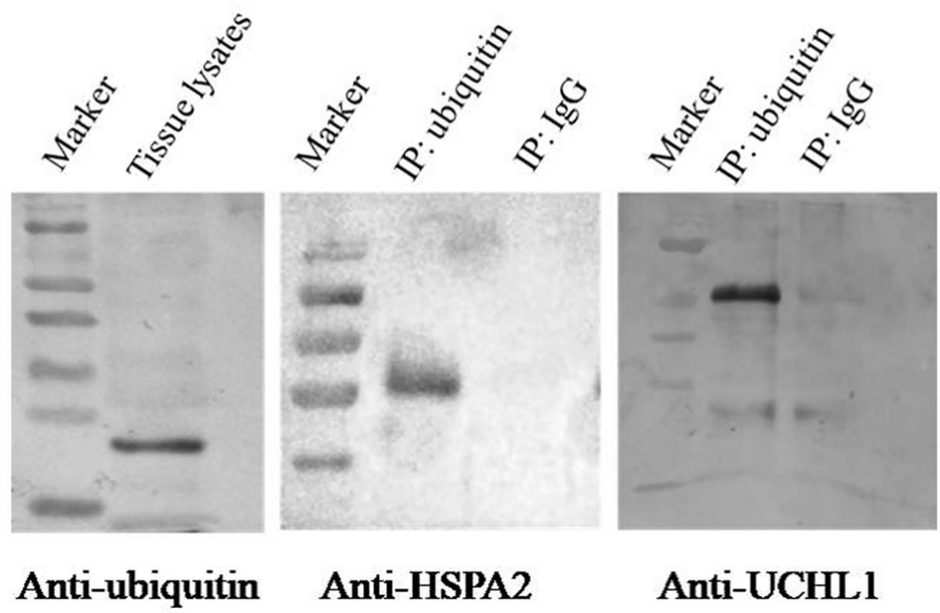
Verification of lysine ubiquitination of HSPA2 and UCHL1 in adult buffalo testis Tissue lysates were immunoprecipitated by ubiquitin antibody and the control (IgG) and the ubiquitinated proteins HSPA2 and UCHL1 were presented in the ubiquitin-IP pulldown compared with IgG control by Western blot analysis.

The results of IP/Western blot analysis of two proteins (UCHL1 and HSPA2) showed that all of the proteins were presented in the ubiquitin-IP pulldown compared with IgG control (Figure 7). UCHL1 and HSPA2 may relate to spermatogenesis according to the above bioinformatics analysis (Figure 6, Supplementary Table S11). We further confirmed the lysine ubiquitination of these proteins.

## Discussion

In the present study, we demonstrate the enrichment of endogenous ubiquitylated peptides from buffalo testicular tissue lysates and their subsequent identification by MS. The presented dataset contains 422 ubiquitylation sites corresponding to 262 lysine ubiquitinated proteins and by far the first ubiquitome in the adult buffalo testis. Recently, immunoaffinity reagents have been reported that are capable of capturing K-GG peptides from ubiquitin and its thousands of cellular substrates. A limitation of this approach is that it does not distinguish between various types of modifications, such as monoubiquitination, polyubiquitination, and the rare NEDD8 and ISG15 modifications [36,37]. However, expression of NEDD8 in mammalian tissues was shown to be developmentally down-regulated [38], and ISG15 expression in bovine tissues is low in the absence of interferon stimulation [39]. In cell culture experiments it was shown that a great majority of sites identified using di-glycine-lysine-specific antibodies stems from ubiquitylated peptides [36]. Thus, most di-Gly remnants derived from cellular peptides are derived from ubiquitinated proteins. Therefore, in the present study we refer to all di-Gly modified lysines as ‘ubiquitination sites’, even though a small portion of these sites might be derived from an NEDD8 or an ISG15 modification.

Until recently, the ubiquitome was only reported in mammalian cells and tissues in human and murine [16–22]. Comparison with the mUbiSiDa (database of mammalian protein ubiquitination site) [40] revealed that 216 sites were common with the database, and 206 were newly identified (Supplementary Figure S2 and Table S12). Further analysis demonstrated that most of the common proteins were involved in metabolic process, localization, transport, and catabolic (Supplementary Table S12). This indicated that ubiquitination is conserved in mammalian and plays important roles in metabolic process. While most of the newly identified proteins in buffalo testis were associated with cellular process, metabolic process, reproduction, and protein complex assembly. Besides, reproduction-related processes such as reproduction, organelle fission, spermatogenesis, and fertilization were only found in buffalo testis (Supplementary Table S12). It may be due to the first detection of whole ubiquitome in mammalian testis. In a word, the results imply that lysine ubiquitination plays both common and specific roles in different cells or tissues in different mammalian species.

Ubiquitination has been established to be an important PTM process regulating protein function in diverse physiological aspects of the eukaryotic cells [41]. Our results demonstrated that the ubiquitinated proteins widely distributed in various cell components and involved in a variety of biological processes. The mammalian testis consists of two compartments, the interstitium and seminiferous tubules, of which the seminiferous tubules account for approximately 82% of total testis volume in buffalo testis and the important site of spermatogenesis [42,43]. In other words, spermatogenesis is the most important function of the testis. Hence, we focus on meiotic, fertilization, and spermatogenesis associated proteins. Here, four proteasome subunits including proteasome activator complex subunit 4 (PSME4), proteasome 26S subunit ATPase 3 (PSMC3), 26S proteasome non-ATPase regulatory subunit 13 (PSMD13), and proteasomal ubiquitin receptor ADRM1 were involved in proteasome pathway (Supplementary Figure S3). PSME4, also called PA200, is a proteasome activator and widely expressed in mammalian tissue especially in testes. It may be involved in the repair of DNA double-strand breaks and PSME4-deficient mice exhibited a marked reduction in male fertility due to defects in pre- and postmeiosis in spermatogenesis [44,45]. PSME4 was also found in the mouse epididymis sperm proteome a few years ago [46], nonetheless, in our study, we first analyzed one lysine ubiquitination site (K918) on PSME4 that may be critical for spermatid development. PSMC3, PSMD13, and ADRM1 are 19S regulatory particles of the 26S proteasome in proteasome pathway. PSMC3 interacts with PSME4 in the protein interaction network and was ubiquitinated on K54 in buffalo testis. It is noteworthy that PSMC3 was found in acrosome membranes during rat spermatid development [47]. PSMD13 was related to meiosis I process in GO analysis and was ubiquitinated on two lysine sites (K84, K130) in buffalo testis. ADRM1, also termed as Rpn13 or GP110, is a novel proteasomal ubiquitin receptor that directly binds to deubiquitinating enzyme UCH37 and promotes its activity [48]. Adult Rpn13^−/−^ mice were infertile due to defective gametogenesis [49]. It is possible that ADRM1 K21 ubiquitination might be very important for buffalo spermatogenesis in our study.

Ubiquitin carboxyl-terminal hydrolases USP14, UCHL1, and CYLD were ubiquitylated on K214, K4, and K532 respectively in our research. USP14 protein was widely distributed in the cytoplasm of round and elongating spermatids in mice testes and USP14-deficient testes revealed the presence of degenerating germ cells and abnormal spermatogenesis [50]. UCHL1 was detected in mouse testicular spermatogonia and Sertoli cells and played a special role in mitotic proliferation and differentiation of spermatogonial stem cells [51–53]. To further explore the potential functions of UCHL1 in spermatogenesis, we investigated by immunohistochemistry analysis in pre-puberal and adult buffalo testes. UCHL1 was mainly localized in the cytoplasm and membrane of spermatogonia in pre-puberal and adult buffalo testes, while the expression quantity decreased with age (Supplementary Figure S4). In the present study, we found that UCHL1 was ubiquitylated on K4 and verified by ubiquitin-IP coupled with Western blot analysis (Figure 7), which may be one of the reasons for the decreased expression of UCHL1 in adult buffalo testes. CYLD, another pivotal deubiquitinating enzyme, performed an essential function in regulation of spermatogenesis and male fertility. CYLD knockout male mice were sterile and revealed severe abnormalities in the seminiferous tubules and further caused impaired spermatogenesis [13]. Thus, the three deubiquitinating enzymes in ubiquitin proteasome system are required during spermatogenesis in buffalo. In addition, E3 ubiquitin protein ligase HUWE1 was identified to mediate ubiquitination of core histones and highly expressed in nuclei from spermatogonia to mid-pachytene spermatocytes in testis and may play a role in chromatin modification in early germ cells [54,55]. Ubiquilin 1 (UBQLN1), interacts with sperm maturation 1 (SPEM1), localized to the manchette of elongating spermatid and participates in spermiogenesis [56].

We found that three heat shock proteins including HSP90AA1, HSPA4, and HSPA2 were ubiquitylated and may related to spermatogenesis process according to bioinformatics analysis. The molecular chaperone HSP90 is ubiquitous highly conserved in eukaryotes and has two isoforms, HSP90AA1 and HSP90AB1. The mutant HSP90 alleles in male *Drosophila melanogaster* are sterile and display meiotic disruption [57]. In male mice, the absence of HSP90AA1 lead to spermatogenesis arrests specifically at the pachytene stage of meiosis I in spite of the development of the reproductive system appears to be normal [58]. In the present study, we found that ubiquitination occurred at six lysine residues (K58, K84, K69, K185, K328, and K420) of HSP90AA1. Proteomics analysis revealed that there were 39 and 46 ubiquitination sites in human HSP90AA1 and HSP90AB1, respectively [59]. We speculated that the comprehensive ubiquitination of HSP90AA1 might associate with spermatogenesis and other biological processes in buffalo testis. HSPA4 and HSPA2 belong to the heat shock protein 70 family, and HSPA4 binds to HSP90AA1 in protein–protein interaction network (Figure 6). It is reported that HSPA4 is expressed in both germ cells and Sertoli cells, and HSPA4-deficient male mice display impaired fertility due to the drastically reduction in the number of spermatozoa and their motility [60]. We first found that one ubiquitylated site K686 in HSPA4 and it may require for normal spermatogenesis. The molecular chaperone HSPA2 has been verified to play a critical role in meiosis, spermatogenesis, male fertility, and sperm-oocyte binding [61–65]. In our previous study, we have identified HSPA2 protein in buffalo testicular seminiferous tubules and suggested that HSPA2 may participate in buffalo spermatogenesis [14]. Here, we further discovered that two ubiquitination sites K127 and K129 in HSPA2 and the lysine ubiquitination confirmed by IP/Western blot analysis (Figure 7). It is speculated that the lysine ubiquitination of HSPA2 may play an important role in reproduction processes.

## Conclusion

In conclusion, the present study is the first proteome-wide analysis of ubiquitylated proteins in the adult buffalo testis. We identified a total of 422 ubiquitination sites from 262 proteins using an integrated proteomics approach. Our findings indicate that lysine ubiquitination may play a critical role in a broad range of cellular processes especially in spermatogenesis. Thus, we speculated that the ubiquitome widens the range of physiological process regulation and provides a rich resource to examine the functions of lysine ubiquitylated in testis in buffalo and mammals. Further studies are needed to verify the functions of these ubiquitylated proteins and elucidate the regulatory roles and mechanisms of lysine ubiquitination in buffalo testis.

## Supplementary Material

Supplementary Figures S1-S4

Supplementary Tables S1-S12

## Abbreviations

di-Gly: diglycine
GO: Gene Ontology
HSPA2: heat shock related 70 kDa protein 2
IP: immunoprecipitation
KEGG: Kyoto Encyclopedia of Genes and Genomes
MS: mass spectrometry
MS/MS: tandem mass spectrometry
PSMC3: proteasome 26S subunit ATPase 3
PSMD13: proteasome non-ATPase regulatory subunit 13
PSME4: proteasome activator complex subunit 4
Ub: ubiquitin
UCHL1: ubiquitin carboxyl-terminal hydrolase isozyme L1
UPS: ubiquitin–proteasome system

## References

[1] Pickart C.M. and Eddins M.J. (2004) Ubiquitin: structures, functions, mechanisms. Biochim. Biophys. Acta Mol. Cell Res. 1695, 55–72 10.1016/j.bbamcr.2004.09.019 15571809

[2] Hochstrasser M. (1995) Ubiquitin, proteasomes, and the regulation of intracellular protein degradation. Curr. Opin. Cell Biol. 7, 215–223 10.1016/0955-0674(95)80031-X 7612274

[3] Ulrich H.D. and Helen W. (2010) Ubiquitin signalling in DNA replication and repair. Nat. Rev. Mol. Cell Biol. 11, 479–489 10.1038/nrm2921 20551964

[4] Steven B. and Stefan J. (2009) Principles of ubiquitin and SUMO modifications in DNA repair. Nature 458, 461–467 19325626 10.1038/nature07963

[5] Jackson S.P. and Jiri B. (2009) The DNA-damage response in human biology and disease. Nature 461, 1071–1078 10.1038/nature08467 19847258 PMC2906700

[6] Chen Z.J. and Sun L.J. (2009) Nonproteolytic functions of ubiquitin in cell signaling. Mol. Cell 33, 275–286 10.1016/j.molcel.2009.01.014 19217402

[7] Caroline G., Koraljka H. and Ivan D. (2011) The spatial and temporal organization of ubiquitin networks. Nat. Rev. Mol. Cell Biol. 12, 295–307 21448225 10.1038/nrm3099 PMC3654194

[8] Nakamura N. (2013) Ubiquitination regulates the morphogenesis and function of sperm organelles. Cells 2, 732–750 10.3390/cells2040732 24709878 PMC3972651

[9] Cong-Cong H. and Wan-Xi Y. (2013) New insights to the ubiquitin-proteasome pathway (UPP) mechanism during spermatogenesis. Mol. Biol. Rep. 40, 3213–3230 23268313 10.1007/s11033-012-2397-y

[10] Sutovsky P. (2003) Ubiquitin-dependent proteolysis in mammalian spermatogenesis, fertilization, and sperm quality control: killing three birds with one stone. Microsc. Res. Tech. 61, 88–102 10.1002/jemt.10319 12672125

[11] Kopanja D., Roy N., Stoyanova T., Hess R.A., Bagchi S. and Raychaudhuri P. (2011) Cul4A is essential for spermatogenesis and male fertility. Dev. Biol. 352, 278–287 10.1016/j.ydbio.2011.01.028 21291880 PMC3065526

[12] Paul-Albert K., Nicholls P.K., Schmidt F.I., Masatoshi H., Takeshi M., Frydman G.H. et al. (2014) The E2 ubiquitin-conjugating enzyme UBE2J1 is required for spermiogenesis in mice. J. Biol. Chem. 289, 34490–34502 25320092 10.1074/jbc.M114.604132 PMC4263858

[13] Wright A., Reiley W.W., Chang M., Jin W., Lee A.J., Zhang M. et al. (2007) Regulation of early wave of germ cell apoptosis and spermatogenesis by deubiquitinating enzyme CYLD. Dev. Cell 13, 705–716 10.1016/j.devcel.2007.09.007 17981138

[14] Huang Y.L., Fu Q., Pan H., Chen F.M., Zhao X.L., Wang H.J. et al. (2016) Spermatogenesis-associated proteins at different developmental stages of buffalo testicular seminiferous tubules identified by comparative proteomic analysis. Proteomics 16, 2005–2018 10.1002/pmic.201500547 27173832

[15] Junmin P., Daniel S., Elias J.E., Thoreen C.C., Dongmei C., Gerald M. et al. (2003) A proteomics approach to understanding protein ubiquitination. Nat. Biotechnol. 21, 921–926 12872131 10.1038/nbt849

[16] David M., Xiaorong W., Lan H. and Peter K. (2008) Quantitative analysis of global ubiquitination in HeLa cells by mass spectrometry. J. Proteome Res. 7, 4566–4576 18781797 10.1021/pr800468j PMC2758155

[17] Danielsen J.M., Sylvestersen K.B., Bekker-Jensen S., Szklarczyk D., Poulsen J.W., Horn H. et al. (2011) Mass spectrometric analysis of lysine ubiquitylation reveals promiscuity at site level. Mol. Cell. Proteomics 10, M110.003590 10.1074/mcp.M110.003590 21139048 PMC3047152

[18] Shi Y., Chan D.W., Jung S.Y., Malovannaya A., Wang Y. and Qin J. (2011) A data set of human endogenous protein ubiquitination sites. Mol. Cell. Proteomics 10, M110.002089 10.1074/mcp.M110.002089 PMC3098580 20972266

[19] Guoqiang X., Paige J.S. and Jaffrey S.R. (2010) Global analysis of lysine ubiquitination by ubiquitin remnant immunoaffinity profiling. Nat. Biotechnol. 28, 868–873 20639865 10.1038/nbt.1654 PMC2946519

[20] Wagner S.A., Beli P., Weinert B.T., Nielsen M.L., Cox J., Mann M. et al. (2011) A proteome-wide, quantitative survey of in vivo ubiquitylation sites reveals widespread regulatory roles. Mol. Cell. Proteomics 10, M111.013284 10.1074/mcp.M111.013284 PMC3205876 21890473

[21] Huttlin E.L. (2011) Systematic and quantitative assessment of the ubiquitin-modified proteome. Mol. Cell 44, 325–340 21906983 10.1016/j.molcel.2011.08.025 PMC3200427

[22] Wagner S.A., Beli P., Weinert B.T., Schölz C., Kelstrup C.D., Young C. et al. (2012) Proteomic analyses reveal divergent ubiquitylation site patterns in murine tissues. Mol. Cell. Proteomics 11, 1578 22790023 10.1074/mcp.M112.017905 PMC3518112

[23] Sharma A.K. and Gupta R.C. (1980) Duration of seminiferous epithelial cycle in buffalo bulls (Bubalus bubalis). Anim. Reprod. Sci. 3, 217–224 10.1016/0378-4320(80)90018-4

[24] Pawar H.S. and Wrobel K.H. (1991) Quantitative aspects of water buffalo (*Bubalus bubalis*) spermatogenesis. Arch. Histol. Cytol. 54, 491–509 1793664 10.1679/aohc.54.491

[25] Vizcaíno J.A., Csordas A., Deltoro N., Dianes J.A., Griss J., Lavidas I. et al. (2016) 2016 update of the PRIDE database and its related tools. Nucleic Acids Res. 44, D447–D456 10.1093/nar/gkv1145 26527722 PMC4702828

[26] Chou M.F. and Schwartz D. (2011) Biological sequence motif discovery using motif-x. Curr. Protoc. Bioinformatics 35, 15–24 10.1002/0471250953.bi1315s35 21901740

[27] Petersen B., Petersen T.N., Andersen P., Nielsen M. and Lundegaard C. (2009) A generic method for assignment of reliability scores applied to solvent accessibility predictions. BMC Struct. Biol. 9, 51 10.1186/1472-6807-9-51 19646261 PMC2725087

[28] Alex M., Hsin-Yu C., Louise D., Matthew F., Sarah H., Rodrigo L. et al. (2015) The InterPro protein families database: the classification resource after 15 years. Nucleic Acids Res. 43, 213–221 10.1093/nar/gku1243 PMC4383996 25428371

[29] Paul H., Keun-Joon P., Takeshi O., Naoya F., Hajime H., Adams-Collier C.J. et al. (2007) WoLF PSORT: protein localization predictor. Nucleic Acids Res. 35, W585–W587 10.1093/nar/gkm259 17517783 PMC1933216

[30] Huntley R.P., Tony S., Prudence M.M., Aleksandra S., Carlos B., Martin M.J. et al. (2015) The GOA database: Gene Ontology annotation updates for 2015. Nucleic Acids Res. 43, 1057–1063 10.1093/nar/gku1113 PMC4383930 25378336

[31] Yuanwei Z., Liangwen Z., Bo X., Yifan Y., Rongjun B., Jun Z. et al. (2013) SpermatogenesisOnline 1.0: a resource for spermatogenesis based on manual literature curation and genome-wide data mining. Nucleic Acids Res. 41, D1055 23193286 10.1093/nar/gks1186 PMC3531227

[32] Kanehisa M. and Goto S. (2000) KEGG: Kyoto Encyclopaedia of Genes and Genomes. Nucleic Acids Res. 28, 27–30 10.1093/nar/28.1.27 10592173 PMC102409

[33] Xiaoli J., Sherman B.T., Wei H.D., Robert S., Baseler M.W., Lane H.C. et al. (2012) DAVID-WS: a stateful web service to facilitate gene/protein list analysis. Bioinformatics 28, 1805–1806 22543366 10.1093/bioinformatics/bts251 PMC3381967

[34] Tay A.P., Pang C., Winter D.L. and Wilkins M.R. (2017) PTMOracle: a Cytoscape app for covisualizing and coanalyzing post-translational modifications in protein interaction networks. J. Proteome Res. 16, 1988–2003 10.1021/acs.jproteome.6b01052 28349685

[35] Do-Young K., Mark S., Smith L.M. and Vierstra R.D. (2013) Advanced proteomic analyses yield a deep catalog of ubiquitylation targets in Arabidopsis. Plant Cell 25, 1523–1540 23667124 10.1105/tpc.112.108613 PMC3694690

[36] Kim W., Bennett E.J., Huttlin E.L., Guo A. and Gygi S.P. (2011) Systematic and quantitative assessment of the ubiquitin-modified proteome. Mol. Cell 44, 325–340 10.1016/j.molcel.2011.08.025 21906983 PMC3200427

[37] Bustos D., Bakalarski C.E., Yang Y., Peng J. and Kirkpatrick D.S. (2012) Characterizing ubiquitination sites by peptide-based immunoaffinity enrichment. Mol. Cell. Proteomics 11, 1529–1540 10.1074/mcp.R112.019117 22729469 PMC3518114

[38] Kamitani T., Kito K., Nguyen H.P. and Yeh E.T.H. (1997) Characterization of NEDD8, a developmentally down-regulated ubiquitin-like protein. J. Biol. Chem. 272, 28557–28562 10.1074/jbc.272.45.28557 9353319

[39] Ling Y., Zhang L.-Y., Wang C., Wang B., Wang X.-M. and Zeng S.-M. (2012) Differential expression pattern of ISG15 in different tissue explants and cells induced by various interferons. Microbiol. Immunol. 56, 163–170 22308980 10.1111/j.1348-0421.2012.00419.x

[40] Chen T., Zhou T., He B., Yu H., Guo X., Song X. et al. (2014) mUbiSiDa: a comprehensive database for protein ubiquitination sites in mammals. PLoS ONE 9, e85744 10.1371/journal.pone.0085744 24465676 PMC3894998

[41] Chen Z., Luo X., Lu Y., Zhu T., Wang J., Tsun A. et al. (2013) Ubiquitination signals critical to regulatory T cell development and function. Int. Immunopharmacol. 16, 348–352 10.1016/j.intimp.2013.01.023 23415874

[42] Johnson L., Varner D.D., Roberts M.E., Smith T.L., Keillor G.E. and Scrutchfield W.L. (2000) Efficiency of spermatogenesis: a comparative approach. Anim. Reprod. Sci. 60, 471–480 10.1016/S0378-4320(00)00108-1 10844217

[43] Wrobel K.H. and Schimmel M. (1989) Morphology of the bovine Sertoli cell during the spermatogenetic cycle. Cell Tissue Res. 257, 93–103 2752416 10.1007/BF00221638

[44] Ustrell V., Hoffman L. and Pratt G.M. (2014) PA200, a nuclear proteasome activator involved in DNA repair. EMBO J. 21, 3516–3525 10.1093/emboj/cdf333 PMC126083 12093752

[45] Bernard K., Bredemeyer A.L., Ching-Yu H., Turnbull I.R., Ryan E., Maggi L.B. et al. (2006) Proteasome activator PA200 is required for normal spermatogenesis. Mol. Cell. Biol. 26, 2999–3007 16581775 10.1128/MCB.26.8.2999-3007.2006 PMC1446934

[46] Skerget S., Rosenow M.A., Petritis K. and Karr T.L. (2015) Sperm proteome maturation in the mouse epididymis. PLoS ONE 10, e0140650 10.1371/journal.pone.0140650 26556802 PMC4640836

[47] Rivkin E., Kierszenbaum A.L., Gil M. and Tres L.L. (2010) Rnf19a, a ubiquitin protein ligase, and Psmc3, a component of the 26S proteasome, tether to the acrosome membranes and the head-tail coupling apparatus during rat spermatid development. Dev. Dyn. 238, 1851–1861 10.1002/dvdy.22004 19517565

[48] Qiu X.B., Ouyang S.Y., Li C.J., Miao S., Wang L. and Goldberg A.L. (2014) hRpn13/ADRM1/GP110 is a novel proteasome subunit that binds the deubiquitinating enzyme, UCH37. EMBO J. 25, 5742–5753 10.1038/sj.emboj.7601450 PMC1698896 17139257

[49] Amin A.S., Jhaver K.G., Peter V., Carrie W., Juliane H., Davis J.J. et al. (2010) Regulators of the proteasome pathway, Uch37 and Rpn13, play distinct roles in mouse development. PLoS ONE 5, e13654 21048919 10.1371/journal.pone.0013654 PMC2965108

[50] Crimmins S., Sutovsky M., Chen P.C., Huffman A., Wheeler C., Swing D.A. et al. (2009) Transgenic rescue of ataxia mice reveals a male-specific sterility defect. Dev. Biol. 325, 33–42 10.1016/j.ydbio.2008.09.021 18926813 PMC2651617

[51] Jungkee K., Yu-Lai W., Rieko S., Satoshi S., Mikako S., Yae S. et al. (2004) Developmental regulation of ubiquitin C-terminal hydrolase isozyme expression during spermatogenesis in mice. Biol. Reprod. 71, 515–521 15084487 10.1095/biolreprod.104.027565

[52] Kwon J., Mochida K., Wang Y.L., Sekiguchi S., Sankai T., Aoki S. et al. (2005) Ubiquitin C-terminal hydrolase L-1 is essential for the early apoptotic wave of germinal cells and for sperm quality control during spermatogenesis. Biol. Reprod. 73, 29–35 10.1095/biolreprod.104.037077 15744022

[53] Wang Y.L., Liu W., Sun Y.J., Kwon J., Setsuie R., Osaka H. et al. (2010) Overexpression of ubiquitin carboxyl‐terminal hydrolase L1 arrests spermatogenesis in transgenic mice. Mol. Reprod. Dev. 73, 40–49 10.1002/mrd.20364 16177983

[54] Zhiqian L., Rose O. and Wing S.S. (2005) Characterization of E3Histone, a novel testis ubiquitin protein ligase which ubiquitinates histones. Mol. Cell. Biol. 25, 2819–2831 15767685 10.1128/MCB.25.7.2819-2831.2005 PMC1061639

[55] Liu Z., Miao D., Xia Q., Hermo L. and Wing S.S. (2007) Regulated expression of the ubiquitin protein ligase, E3 Histone /LASU1/Mule/ARF-BP1/HUWE1, during spermatogenesis. Dev. Dyn. 236, 2889–2898 10.1002/dvdy.21302 17823942

[56] Bao J., Zhang J., Zheng H., Xu C. and Yan W. (2015) UBQLN1 interacts with SPEM1 and participates in spermiogenesis. Mol. Cell. Endocrinol. 327, 89–97 10.1016/j.mce.2010.06.006 PMC2950875 20558241

[57] Yue L., Karr T.L., Nathan D.F., Swift H., Srinivasan S. and Lindquist S. (1999) Genetic analysis of viable Hsp90 alleles reveals a critical role in Drosophila spermatogenesis. Genetics 151, 1065–1079 10049923 10.1093/genetics/151.3.1065 PMC1460532

[58] Iwona G., Cederroth C.R., Walicki J., Corinne G., Sofia B., Nicolas W. et al. (2010) The molecular chaperone Hsp90α is required for meiotic progression of spermatocytes beyond pachytene in the mouse. PLoS ONE 5, e15770 21209834 10.1371/journal.pone.0015770 PMC3013136

[59] Mayer M. and Lebreton L. (2015) Hsp90: breaking the symmetry. Mol. Cell 58, 8–20 10.1016/j.molcel.2015.02.022 25839432

[60] Held T., Barakat A.Z., Mohamed B.A., Paprotta I., Meinhardt A., Engel W. et al. (2011) Heat-shock protein HSPA4 is required for progression of spermatogenesis. Reproduction 142, 133–144 10.1530/REP-11-0023 21487003

[61] Dix D.J., Allen J.W., Collins B.W., Poorman-Allen P., Mori C., Blizard D.R. et al. (1997) HSP70-2 is required for desynapsis of synaptonemal complexes during meiotic prophase in juvenile and adult mouse spermatocytes. Development 124, 4595 9409676 10.1242/dev.124.22.4595

[62] Govin J., Caron C., Escoffier E., Ferro M., Kuhn L., Rousseaux S. et al. (2006) Post-meiotic shifts in HSPA2/HSP70.2 chaperone activity during mouse spermatogenesis. J. Biol. Chem. 281, 37888–37892 10.1074/jbc.M608147200 17035236 PMC1896149

[63] Christian R., Anna U., Michael H., Simon A., Preethi V., Diana B. et al. (2014) HSP70-binding protein HSPBP1 regulates chaperone expression at a posttranslational level and is essential for spermatogenesis. Mol. Biol. Cell 25, 2260–2271 24899640 10.1091/mbc.E14-02-0742 PMC4116300

[64] Bromfield E.G., McLaughlin E.A., Aitken R.J. and Nixon B. (2016) Heat Shock Protein member A2 forms a stable complex with angiotensin converting enzyme and protein disulfide isomerase A6 in human spermatozoa. Mol. Hum. Reprod. 22, 93 10.1093/molehr/gav073 26676989

[65] Bromfield E.G., Aitken R.J., Anderson A.L., McLaughlin E.A. and Brett N. (2015) The impact of oxidative stress on chaperone-mediated human sperm-egg interaction. Hum. Reprod. 30, 2597–2613 10.1093/humrep/dev214 26345691

