# Supplementary material for: Ubiquitome analysis reveals the involvement of lysine ubiquitination in the spermatogenesis process of adult buffalo (Bubalus bubalis) testis
Source: Biosci Rep. 2020 Jun 16;40(6):BSR20193537. doi: 10.1042/BSR20193537 (PMC7298129; doi:10.1042/BSR20193537)
Supplement: Supplementary Figures S1-S4 [file BSR-2019-3537_supp.pdf]

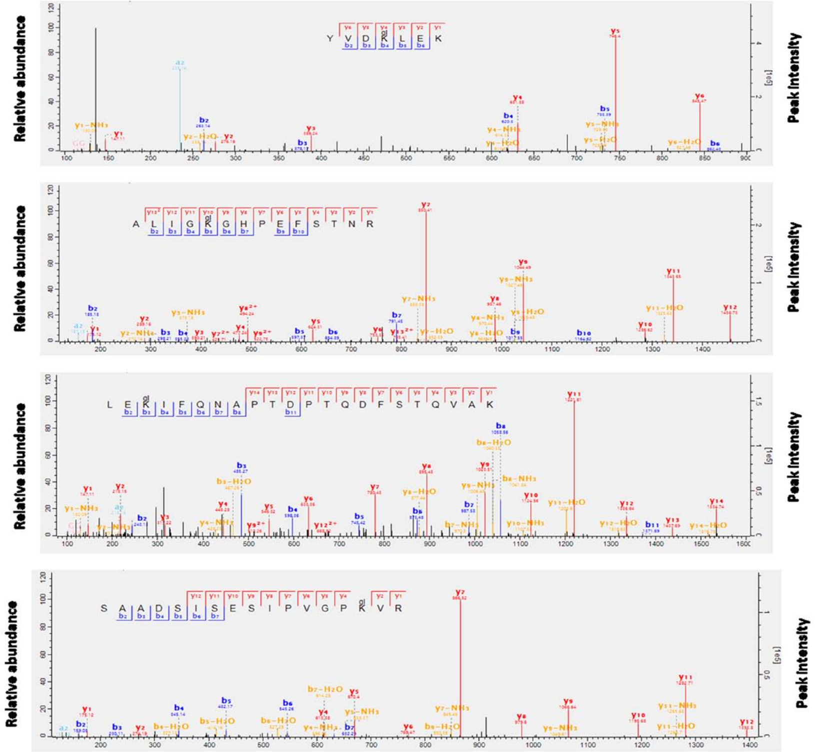
Relative abundance
Peak Intensity
[1e5]
Y V D K L E K
gl
A L I G K G H P E F S T N R
gl
L E K I F Q N A P T D P T Q D F S T Q V A K
gl
S A A D S I S E S I P V G P K V R
gl

Supplementary Fig.S1. The MS/MS spectra of the representative ubiquitinated peptides from ubiquitin carboxyl-terminal hydrolase (USP5). The spectra are shown for ubiquitination sites of K357, K423, K360 and K793, respectively, and their peptide sequences are shown in the respective spectrum.

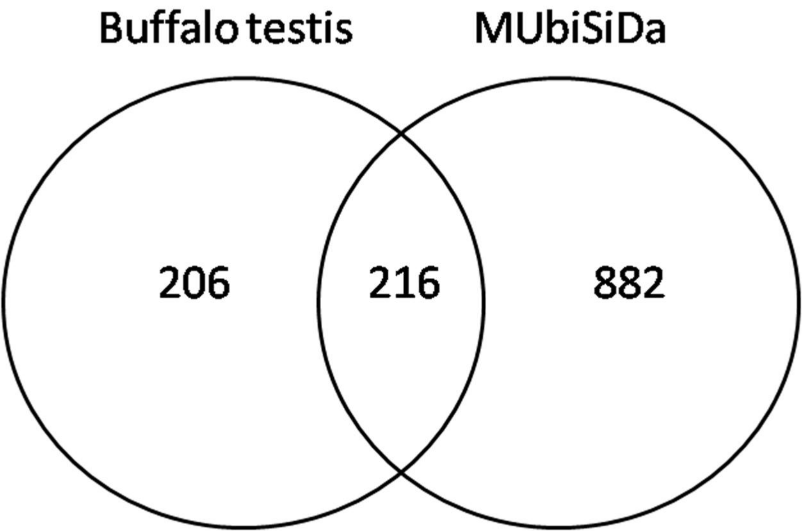
Buffalo testis
MUbiSiDa
206
216
882

Supplementary Fig.S2. The Venn diagram for ubiquitination sites of buffalo testis compared to the mUbiSiDa database.

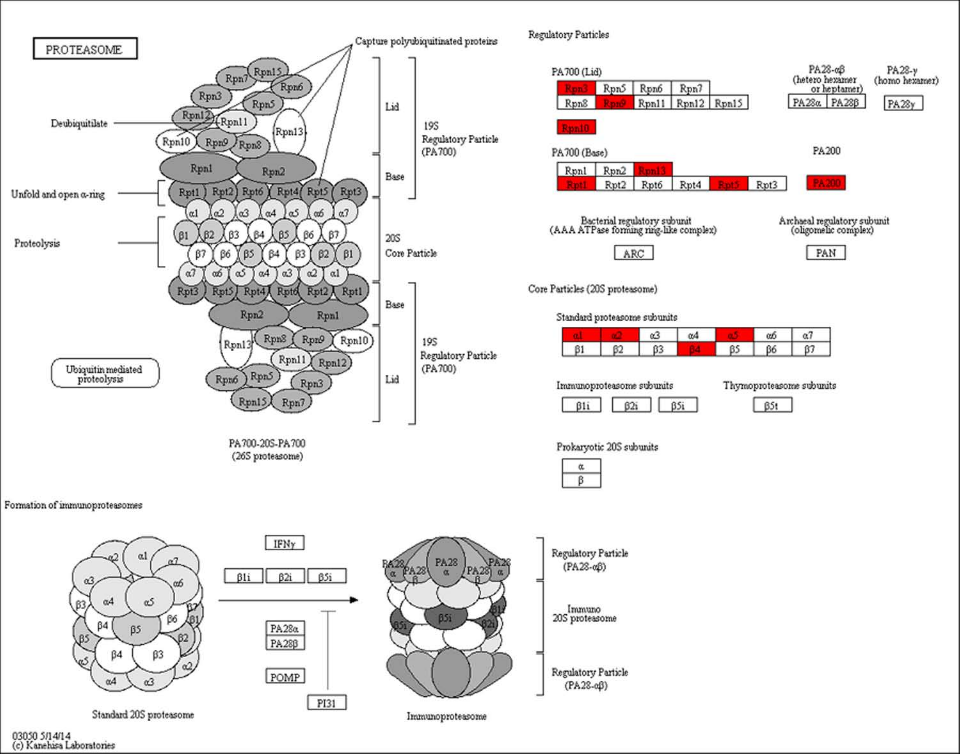

PROTEASOME
Capture polyubiquitinated proteins
Deubiquitilate
Unfold and open α-ring
Proteolysis
Rpn15
Rpn7
Rpn6
Rpn3
Rpn5
Rpn12
Rpn11
Rpn13
Rpn10
Rpn9
Rpn8
Rpn1
Rpn2
Rpt1 Rpt2 Rpt6 Rpt4 Rpt5 Rpt3
α1 α2 α3 α4 α5 α6 α7
β1 β2 β3 β4 β5 β6 β7
β7 β6 β5 β4 β3 β2 β1
α7 α6 α5 α4 α3 α2 α1
Rpt3 Rpt5 Rpt4 Rpt6 Rpt2 Rpt1
Lid
Base
19S Regulatory Particle (PA700)
20S Core Particle
Ubiquitin mediated proteolysis
PA700-20S-PA700 (26S proteasome)
Regulatory Particles
PA700 (Lid)
Rpn3 Rpn5 Rpn6 Rpn7
Rpn8 Rpn9 Rpn11 Rpn12 Rpn15
Rpn10
PA28-αβ (hetero hexamer or heptamer)
PA28α PA28β
PA28-γ (homo hexamer)
PA28γ
PA700 (Base)
Rpn1 Rpn2 Rpn13
Rpt1 Rpt2 Rpt6 Rpt4 Rpt5 Rpt3
PA200
Bacterial regulatory subunit (AAA ATPase forming ring-like complex)
ARC
Archaeal regulatory subunit (oligomeric complex)
PAN
Core Particles (20S proteasome)
Standard proteasome subunits
α1 α2 α3 α4 α5 α6 α7
β1 β2 β3 β4 β5 β6 β7
Immunoproteasome subunits
β1i β2i β5i
Thymoproteasome subunits
β5t
Prokaryotic 20S subunits
α
β
Formation of immunoproteasomes
IFNγ
β1i β2i β5i
PA28α
PA28β
POMP
PI31
Standard 20S proteasome
Immunoproteasome
Regulatory Particle (PA28-αβ)
Immuno 20S proteasome
Regulatory Particle (PA28-αβ)
03050 5/14/14
(c) Kanehisa Laboratories

Supplementary Fig.S3. KEGG pathway enrichment analysis of the ubiquitinated proteins in proteasome. The ubiquitinated proteins are highlighted in red.

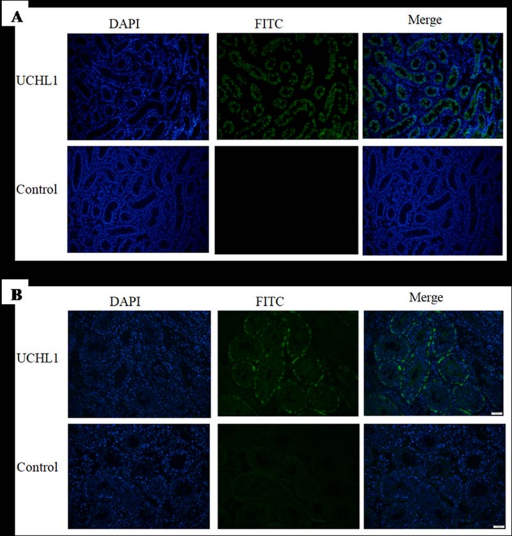

A
DAPI
FITC
Merge
UCHL1
Control
B
DAPI
FITC
Merge
UCHL1
Control

Supplementary Fig.S4. Immunohistochemical analyses of UCHL1 in pre-puberal (A) and adult (B) buffalo testes. UCHL1 which is mainly localized in the cytoplasm and membrane of spermatogonia in pre-puberal and adult buffalo testes, while the expression quantity decreased with age. The negative controls were incubated in blocking solution without the primary antibody. Scale bars=100 μm.
